# Transcriptome Study of an *Exophiala dermatitidis PKS1* Mutant on an *ex Vivo* Skin Model: Is Melanin Important for Infection?

**DOI:** 10.3389/fmicb.2018.01457

**Published:** 2018-07-03

**Authors:** Caroline Poyntner, Ursula Mirastschijski, Katja Sterflinger, Hakim Tafer

**Affiliations:** ^1^Department of Biotechnology, VIBT EQ Extremophile Center, University of Natural Resources and Life Sciences, Vienna, Austria; ^2^Wound Repair Unit, Center for Biomolecular Interactions Bremen, University of Bremen, Bremen, Germany; ^3^Division of Plastic and Aesthetic Surgery, Rotkreuzklinikum München, Munich, Germany

**Keywords:** melanin, black yeast, *Exophiala dermatitidis*, *PKS1*, skin model, virulence

## Abstract

The black yeast *Exophiala dermatitidis* is a polyextremophilic human pathogen, especially known for growing in man-made extreme environments. Reported diseases caused by this fungus range from benign cutaneous to systemic infections with 40% fatality rate. While the number of cases steadily increases in both immunocompromised and immunocompetent people, detailed knowledge about infection mechanisms, virulence factors and host response are scarce. To understand the impact of the putative virulence factor melanin on the infection, we generated a polyketide synthase (*PKS1*) mutant using CRISPR/Cas9 resulting in a melanin deficient strain. The mutant and the wild-type fungus were inoculated onto skin explants using an *ex vivo* skin organ culture model to simulate *in vivo* cutaneous infection. The difference between the mutant and wild-type transcriptional landscapes, as assessed by whole RNA-sequencing, were small and were observed in pathways related to the copper homeostasis, cell wall genes and proteases. Seven days after inoculation the wild-type fungus completely colonized the stratum corneum, invaded the skin and infected keratinocytes while the mutant had only partially covered the skin and showed no invasiveness. Our results suggest that melanin dramatically improves the invasiveness and virulence of *E. dermatitidis* during the first days of the skin infection.

## Introduction

The Ascomycete *Exophiala dermatitidis* belongs to the group of black yeasts, a heterogeneous taxonomic and phylogenetic group that shares melanized cell walls and yeast-like budding as common features (Sterflinger, 2006). Black yeasts are known for their exceptional abilities to survive extreme environments e.g., salterns (Gunde-Cimerman et al., 2000) and glaciers (Branda et al., 2010) but also man-made extreme environments e.g., sauna facilities (Matos et al., 2002) and dishwashers (Döğen et al., 2013b). In humans, *E. dermatitidis* colonization is often reported in cystic fibrosis patients with a prevalence rate varying from 4.8 to 15.7% in Germany and Belgium (Pihet et al., 2009). Cutaneous and subcutaneous abscesses (Zeng et al., 2007), septic arthritis, endocarditis, catheter-associated fungemia (Byrne and Reboli, 2017) but also systemic infections have been reported (Hiruma et al., 1993). A huge difference is observed in etiology and severity (Sudhadham et al., 2008). Theories to explain these differences exist (Matos et al., 2002; Poyntner et al., 2016) but detailed studies about the infection mechanisms are scarce. One of the proposed virulence factors is melanin (Taborda et al., 2008), a negatively charged, hydrophobic macromolecule composed of oxidative polymerized phenolic or indolic monomers and often complexed with proteins and carbohydrates (Butler and Day, 1998; Casadevall et al., 2000). It was shown that melanin protects *Cryptococcus neoformans* against oxidants produced by the host and that its production increases with high pH, illumination with visible light and zinc addition (Wang et al., 1995) and might therefore help the fungus to survive in extreme environments. Melanin confers an increased resistance against the phagolysosomal oxidative burst of human neutrophils (Schnitzler et al., 1999) and antifungal drugs (Paolo et al., 2006). A melanin deficient mutant showed a substantial decrease in virulence and case fatality rate in albino mice but retained its neurotropic potential (Dixon et al., 1987). In this study we sought to assess the impact of melanin on the first phase of fungal skin infection. To this aim we used the CRISPR/Cas9 technology in the black yeast *E. dermatitidis* to knockout the *PKS1* gene, leading to the disruption of the DHN melanin production. The fungal growth of both strains on *ex vivo* skin models was monitored microscopically and macroscopically while differences in gene regulations in the mutant and the wild-type were assessed by total RNA sequencing 7 days after inoculation.

The wild-type caused epidermolysis, ballooned keratinocytes, dermal infiltration and covered the whole skin model, while the mutant partially colonized the epidermis but did not grow invasively. On the transcriptome level only minor changes were observed. Our results indicate that melanin produced by the DHN pathway is important for the invasion. We hypothesize that the fungus might use the ability of human keratinocytes to absorb exogenous melanin in order to introduce fungal melanin and other virulence factors like proteases, ureases and DNases into the keratinocytes like a like a “trojan horse”, which ultimately leads to dermal disintegration.

## Materials and Methods

### CRISPR/Cas9 Editing

As described before by Feng et al. (Feng et al., 2001) a mutation of the *PKS1* gene leads into a melanin deficient strain. Based on this study we used sgRNA scorer 2 (Chari et al., 2017) to find single guided RNA (sgRNA) targets against the gene HMPREF1120_03173 on both strands. We selected the five highest scoring targets mapping 5′ half of the gene (**Supplementary Table S1**). The corresponding sgRNAs and Cas9 protein were provided by the BCF Protein Technologies Facility, CRISPR Lab, Vienna Biocenter Core Facility^1^.

*Exophiala dermatitidis* (CBS 525.76) was cultivated in YPD broth (2% peptone, 1% bacto yeast extract, and 2% dextrose) for 24 h at room temperature. Cells were counted in a hemocytometer and 10^6^ cells were centrifuged (600 × *g*, 5 min) and chilled on ice for 30 min. The pellet was washed twice with cold 10% glycerol and resuspended in 10% glycerol (200 μl). SgRNA (12 μg), 1x cleavage buffer (10x: 200 mM Hepes, 1.4 M KCl, 5 mM DTT, 100 mM MgCl_2_ and 1 mM EDTA) was concentrated to 5 μl and mixed with Cas9 protein (5 μg, WT Cas9 from *Streptococcus pyogenes* with two nuclear localization signals). The mixture was incubated at 37°C for 10 min. Electroporation was carried out in a Gene Pulser Xcell (Bio-Rad, Hercules, CA, United States) at 2,5 kV field strength, 300 Ω resistance and 25 mF capacitance. Electroporated cells were incubated at 25°C for 3 h, inoculated on YPD media and incubated at 37°C. For each transfection untreated fungal cells were used.

### Cultures With Tricyclazole, L-Dopa, L-Tyrosine

Malt extract agar (2% malt extract, 2% D-glucose, 0.1% bacto-peptone and 2% agar) plates with 50 mg/L tricylazole (LGC Standards, United Kingdom, Li et al., 2016) were inoculated with the wild-type and the mutant strains and incubated at 37°C for 10 days. The tricyclazole served as inhibitor of the pentaketide melanin biosynthesis (DHN pathway) (Franzen et al., 2006). Using a minimal medium (15.0 mM glucose, 10.0 mM MgSO_4_, 29.4 mM KH_2_PO_4_, 13.0 mM glycine, and 3.0 μM thiamin) the mutant and wild-type were also inoculated in liquid cultures with 1 mM L-Dopa (L-Dopa, Merck, Germany) or 1 mM L-tyrosine (Merck, Germany, Paolo et al., 2006). The cultures were kept in the dark to withstand autopolymerisation of L-Dopa at 37°C for 10 days.

### Whole Genome Sequencing of the Mutant

DNA extraction and whole genome sequencing was performed as previously described (Tesei et al., 2017) using the PGM Sequencing platform (Life Technologies, Carlsbad, CA, United States). Sheared genomic DNA (Bioruptor^TM^ CD-200 TS Sonication System, Diagenode, Belgium) were used for the library builder (AB Library Builder^TM^ System, Life Technologies, Carlsbad, CA, United States). The resulting library was quantified (Ion Library TaqMan Quantitation Kit, Life Technologies, Carlsbad, CA, United States) and loaded on the Chef instrument (Life Technologies, Carlsbad, CA, United States) followed by single-end sequencing.

Samtools/Bcftools (Li et al., 2009) and Vcftools (Danecek et al., 2011) were used to identify putative CRISPR/Cas9 mutated sites and SNPeff was used to assess their impacts (Cingolani et al., 2012).

### Skin Culture Experiments

Skin culture experiments were conducted as previously described (Poyntner et al., 2016). Skin for the *ex vivo* skin wound model was obtained from a patient undergoing brachioplasty at Department of Plastic, Reconstructive and Aesthetic Surgery, Klinikum Bremen-Mitte, Germany. The skin was defatted and full-thickness skin explants were cut into pieces with a size of 5 cm × 5 cm. With a scalpel blade, incisional wounds were inflicted on the epidermal surface. Wild-type (14 skin explants, CBS 525.76) or mutant (13 skin explants) were inoculated with a sterile inoculation loop taken from a 7 days old culture grown on 2% malt extract agar. The skin was cultured as described by Mirastschijski et al. (Mirastschijski et al., 2002), with the explants placed at the liquid-air-interface for cultivation of the fungi on top of the skin mimicking pathological conditions. The cultures were kept at 37°C for 7 days and medium was exchanged every second day. A skin control without fungal inoculum was cultured in parallel. For a negative control, wild-type or mutant from a 7 days old MEA culture were inoculated on a prewetted Nylon membrane (0.45 μm, Whatman, Maidstone, Kent, United Kingdom) and incubated with culture medium under the same conditions as the skin model. Biomass was collected for three replicates followed by RNA extraction using the FastRNA Pro RED KIT (MP Biomedicals, Santa Ana, CA, United States).

### RNA Sequencing and Reads Mapping

From the total RNA extracted, mRNA was selected using the Dynabeads mRNA DIRECT Micro Kit (Ambion by Life Technologies, Carlsbad, CA, United States) and the library was constructed using Ion Total RNA-Seq kit v2 (Life Technologies, Carlsbad, CA, United States). Quality and quantity were measured using an Agilent 2100 Bioanalyzer (Agilent Technologies, Santa Clara, CA, United States) and a Qubit 2.0 (Life Technologies, Carlsbad, CA, United States). Targeted library length of 290 bp was selected with a Pippin Prep (Sage Science, Beverly, MA, United States). Sequencing was performed using the Ion Torrent Proton and the HiQ sequencing kit (Life Technologies, Carlsbad, CA, United States). From the sequenced raw data, reads were mapped as described previously (Poyntner et al., 2016). We removed reads originating from human sequences by mapping the total set of reads against the concatenated human (GRCh37) and *E. dermatitidis* genomes with STAR 2.4.1d (Dobin et al., 2013). Reads that mapped against *E. dermatitidis* were used to assess the differentially expressed genes. Reads from the mutant were mapped against the wild-type genome and annotation. Counting of mapped reads on annotation elements was done with featureCounts v1.4.6p2 (Liao et al., 2014) and identification of differentially expressed genes as well as assessment of sample to sample distance was done with R (R Core Team, 2016) and the edgeR package (Robinson et al., 2010). The functional enrichment of the significantly regulated genes was done with GoStat (Falcon and Gentleman, 2007). Revigo (Supek et al., 2011) was used to summarize the lists of overrepresented Gene Ontology terms.

Gene families annotation was obtain end from Poyntner et al. (2016) and Chen et al. (2014).

### Ethics Statement

The skin models were approved by the ethics committee of Bremen (No 336-2012) and all donors signed a written consent prior operation.

### Microscopical Analyses

Skin samples were fixed in formalin, embedded in paraffin and processed as previously described (Mirastschijski et al., 2002) followed by Haematoxylin and Eosin (HE) staining. Microscopic images were taken using an Olympus BX51 microscope.

## Results

### CRISPR/Cas9 Genome Editing

The mutant strain was obtained by using the CRISPR/Cas9 system directed against the gene *PKS1* (HMPREF1120_03173), since its disruption was previously shown to yield melanin deficient *E. dermatitidis* strains (Feng et al., 2001).

We first transfected *E. dermatitidis* with five sgRNAs (2.4 μg each) in one experiment. 20% of the treated cells turned white, while the other fungal cells did not exhibit any change in color. The white fungi however were unstable and changed to light brown after few days. We repeated the experiment sequentially i.e., by using one sgRNA at a time but at a fivefold amount (12 μg, see Material and Methods). Only the sgRNA targeting region 5 (**Supplementary Table S1**) successfully produced a stable melanin deficient strain by effectively knocking out *PKS1* through non-homologous end joining (NHEJ).

**FIGURE 1:**
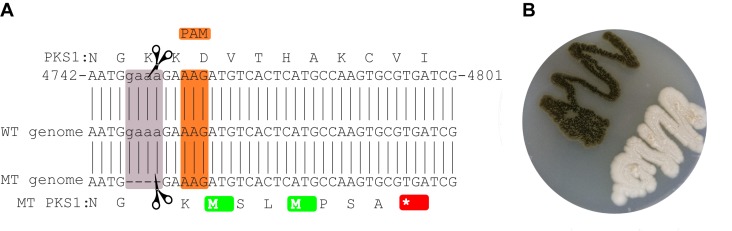
Mutated site in the CRISPR/Cas9-targeted gene. The local PKS1 amino acid and nucleotide sequence in the WT is shown at the top **(A)**. The sequence of the reference genome is shown in the middle, while the assembled sequence of the mutant is shown at the bottom of the figure. The orange and mauve rectangles highlight the PAM and deleted sequences, respectively. The mutation leads to a stop codon 7 amino acids downstream of the deleted region (red). Culture of the mutant and wild-type strains **(B)**.

CRISPR/Cas9 mediated a four nucleotides deletion leading to a frame-shift and a stop codon seven amino acids downstream of the mutation site (**Figure 1**). The deletion site which is located close to a non-canonical PAM sequence (Zhang et al., 2015), does not correspond to any of the regions originally targeted (**Figure 1**). Still the deletion was seen both by PCR and by mapping the reads from the whole genome sequencing of the mutant strain to the wild-type strain The mutation is supported by 33 reads out of the 35 overlapping with the locus (IMF 94.3%).

### Infection Development and Microscopic Observations

**FIGURE 2:**
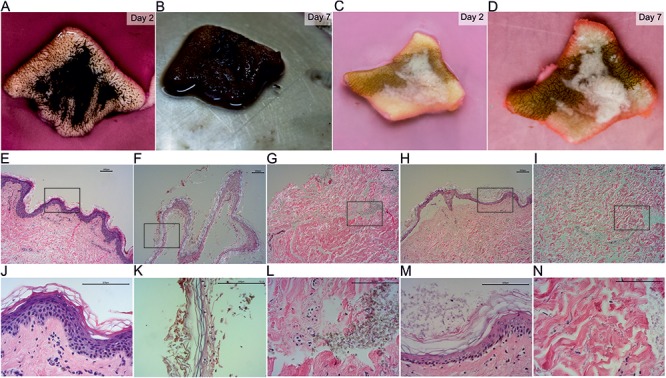
HE stained sections of inoculated *ex vivo* skin explants. *Ex vivo* skin explants after 2 days **(A,C)** and 7 days **(B,D)** inoculated with the wild-type **(A,B)** and the mutant **(C,D)** at 37°C. The brown color in **(C)** and **(D)** derives from surgical ink used for incisional markings in plastic-reconstructive surgery and does not originate from the mutant fungus. HE stained sections of *ex vivo* skin explants without fungal inoculum **(E,J)**, the wild-type **(F,G,K,L)** and the mutant **(H,I,M,N)**. Epidermal samples of the wild-type **(F,K)** and the mutant **(H,M)** and dermal tissue of wild-type **(G,L)** and mutant **(I,N)** are shown. Areas marked with a black box **(E–I)** are shown in higher magnification in the lane below **(J–N)**. Scale bars: 100 μm.

The wild-type was able to grow on the full-thickness skin explants in our *ex vivo* skin wound model. After 7 days, the whole skin surface was covered by fungal biomass (**Figures 2A,B**) similar to previous results (Poyntner et al., 2016). In contrast, the mutant strain partially covered the skin and adhered around the cut (**Figures 2C,D**). By HE histology we found that the skin explants were morphologically intact and vital when cultured without any fungal inoculum under the same conditions of the fungal experiments (**Figures 2E,J**). Skin inoculated with the wild-type showed signs of disintegration with the epidermis separated from the dermis, keratinocytes being pyknotic and increasing epidermal degradation. The fungus changed the color of the stratum corneum (**Figures 2F,K**) and was able to invade and infect the dermis (**Figures 2G,L**). Wild-type cells were bigger in size compared to the mutant. After 7 days the mutant was colonizing the epidermis with growth on top of the stratum corneum. No dermal invasion (**Figures 2I,N**) but attachment to medium exposed surfaces was found (**Figures 2H,M**). No color change of the stratum corneum was visible and only few keratinocytes were pyknotic.

### Whole Genome Sequencing and RNA Sequencing

The genome of the mutant strain was sequenced (see section “Materials and Methods”) resulting in 4247797 reads and 1229354886 sequenced bases. The reads were assembled with Newbler 2.9 into a genome containing 263 contigs, 26351726 bases with a coverage of 46 and a N50 of 198974. This Whole Genome project has been deposited at DDBJ/ENA/Gen Bank under the accession QEYA00000000. The version described in this paper is version QEYA01000000. The WGS reads were uploaded to NCBI (SRR7081810).

The skin infection and control experiments for the wild-type and mutant strains were sequenced in triplicates on the Ion Proton Platform. Between 45 and 75% of the reads were mapping against *E. dermatitidis*, and between 0.9 and 7.3% of them had a human origin. The numbers of sequenced and mapped reads for each experiments are shown in the **Supplementary Table S1**. Principal component analysis of the read counts for each replicate in each experiment shows that the control experiments are well separated from the skin experiments, and that the mutant strain and wild-type strains have a similar transcriptional landscape (**Supplementary Figure S1**).

### Comparison of Enriched Functional Terms in the Set of Regulated Genes in Wild-Type and Mutant

We looked at the differentially expressed genes between the mutant, the wild-type and the wild-type from our previous experiment (Poyntner et al., 2016). For clarity, we name the sequencing data from the mutant experiment as MT, from the current experiment as WT2 and from the previous experiment as WT1.

Two genes were significantly differentially expressed between the WT2 and MT in the control experiment. HMPREF1120_02141 (Gentisate 1, 2 - dioxygenase) and HMPREF1120_02142 (Salicylate hydroxylase). No other genes were significantly regulated between WT and MT in the control and skin experiment.

**FIGURE 3:**
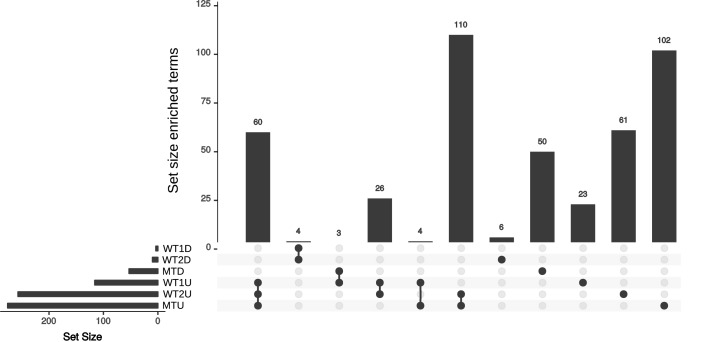
Upset plot for the sets of enriched annotation for the skin experiments. Set of enriched annotations found in the WT1, WT2, and MT. U and D represent the set of enriched annotation for the up- and down-regulated genes, respectively.

Sixty enriched functional terms are shared by the set of upregulated genes in the skin vs. the control for WT1 (**Figure 3**, WT1U), the set of upregulated genes in WT2 (WT2U) and the set of upregulated genes in mutant (MTU). Among those terms, 55 gene ontologies were clustered into three categories (DNA-dependent DNA replication, monocarboxylic acid metabolism and cellular component biogenesis, **Supplementary Table S1**) by Revigo (Supek et al., 2011) indicating an increase in DNA replication and active growth during the skin experiment compared to control. The enrichment of DEAD-Box RNA helicase family and nuclear mRNA exporter (The Transporter Classification Database (TCDB) 3.A.18) indicates an increase in transcriptional activity.

Fifteen gene ontology (GO) terms specific to WT2U and WT1U (**Supplementary Table S1**) are clustered into DNA replication, MCM complex, helicase activity and carbohydrate derivative binding. Accordingly, the enriched protein domains are related to the MCM complex, translation, transcription and large substrate binding. Finally, genes annotated with the Lon Protease S16 domain are enriched in the set of upregulated genes in the WT experiments but not in the MT. Lon proteases were reported to contribute to pathogenicity in fungi (Li et al., 2015) and are known bacterial virulence factors involved in biofilm formation, motility and macrophage survival (Takaya et al., 2003; Breidenstein et al., 2012; Cui et al., 2015). The ensemble of enriched genes in MTU (**Supplementary Table S1**) contains 102 terms, distributed into 91 GO terms, 7 Interpro (IPR) terms and 4 TCDB terms. The TCDB entries are related to the trafficking between the cytoplasm and the mitochondria, while the IPR terms are related to tryptophan synthase, energy homeostasis, translation, secretory proteins and riboflavin synthase. On the GO level, terms related to respiration, translation and transcription indicate that the MT is metabolically active on the *ex vivo* skin samples. The MTD is enriched in terms related to transport (MFS transporter, TCDB *2A1*) and transcription (**Supplementary Table S1**). The group of GO terms were enriched in regulation of biological processes, organic cyclic compound biosynthesis, transmembrane transporter activity and zinc ion binding, indicating that the mutant cells are decreasing their metabolism and transport compared to the control and the WT experiments. This is in line with the slower growth of the MT on the *ex vivo* skin models compared to the WT.

For WT2D and WT1D four terms were enriched: oxidoreductase activity, NAD (P)-binding domain, GroES-like superfamily and alcohol dehydrogenase superfamily (**Supplementary Table S1**).

### Quantitative Analysis

The regulation of genes involved in melanin production, nutrient uptake, metal acquisition, secondary metabolite synthesis and gluconeogenesis was analyzed.

#### Melanin

Three melanin pathways are present in *E. dermatitidis* (Chen et al., 2014) and during skin infection only the L-tyrosine degradation pathway had consistently upregulated genes (Poyntner et al., 2016). This is also seen in the present work in both the WT and MT strains (**Table 1**), where tyrosine aminotransferase and 4-hydroxyphenylpyruvate dioxygenase are upregulated, indicating an increased conversion of L-tyrosine to homogentisate acid, which upon auto-oxidation and polymerization is converted to pyomelanin. In parallel *FahA* (HMPREF1120_03825) and *HmgA* (HMPREF1120_03827), two genes that degrade homogentisate acid to fumarate and acetoacetate, are upregulated (**Table 1** and **Supplementary Table S1**) in WT and MT by at least a factor six. The L-Dopa melanin pathway (Eisenman et al., 2007) is downregulated during growth on skin as was previously reported. Finally, the *Abr1* (Multicopper ferroxidase) component of the DHN melanin pathway is downregulated in WT and MT while *Abr2* (Laccase) is upregulated in all three experiments (**Table 1**).

**Table 1:** List of genes involved in melanin production, nitrogen/carbon/metal acquisition, secondary metabolites production and cell wall regulated either differently in wild-type 1 (WT1), (Poyntner et al., 2016), wild-type 2 (WT2) and mutant (MT) or in all three experiments.

Category	Gene	Homolog	WT1	WT2	MT	Description
DHN melanin pathway	Laccase *Abr2*	HMPREF1120_02828	0.29	0.22	0.18	Fungal pigment *MCO*
	Multicopper fungal ferroxidase *Abr1*	HMPREF1120_04510		42.88	23.29	Fungal ferroxidase
		HMPREF1120_00173			2.55	Ferrooxidoreductase Fet3
Dopa melanin pathway	*melO*	HMPREF1120_07692			0.14	Tyrosinase
		HMPREF1120_03345	0.09	0.17	0.06	
		HMPREF1120_05865	0.29	0.22	0.18	
		HMPREF1120_04578			0.21	
L-tyrosine degradation melanin pathway	*Tat*	HMPREF1120_02164	16.80	32.45	29.45	Tyrosine aminotransferase
	*hppD*	HMPREF1120_05584	4.22	15.67	18.00	4-Hydroxyphenylpyruvate dioxygenase
	*fahA*	HMPREF1120_03825	6.15	9.13	45.25	Fumarylacetoacetate hydrolase
Nitrogen acquisition	*PTR2/POT* transporter	HMPREF1120_06103	0.30	0.13	0.10	*POT* family proton-dependent oligopeptide transporter
		HMPREF1120_02660			0.41	Amino acid transporter
		HMPREF1120_03271	0.26	0.32		Amino acid transporter
	Aspartyle protease	HMPREF1120_05067			0.36	Peptidase
	Cerevisin	HMPREF1120_08439			0.36	*S8* peptidase
	Peptide hydrolase	HMPREF1120_03813			0.23	*M28* peptidase
	Glutamate carboxypeptidase II	HMPREF1120_04355			0.35	*M28* peptidase
Secondary metabolites	*DMATS*	HMPREF1120_01968			3.32	Unknown
	*NRPS*	HMPREF1120_00598	3.76	3.81	2.40	Linear gramicidin synthase subunit C
Fatty-acid beta-oxidation	*FAA2*	HMPREF1120_02478			0.32	Long-chain-fatty-acid–CoA
	*POT1*	HMPREF1120_04123	5.81	8.94	7.11	3-oxoacyl CoA thiolase
Glyoxylate cycle	*MDH1-3/MAE1*	HMPREF1120_06385			2.84	Malate dehydrogenase
		HMPREF1120_0060	3.12	6.65	18.56	Malate dehydrogenase
		HMPREF1120_06787	2.96	2.17	2.81	Malate dehydrogenase
	*Aco1*	HMPREF1120_03751	5.74	15.71		Aconitate hydratase
Gluconeogenesis	*Pyc1*-2	HMPREF1120_06163	5.11	7.83		Pyruvate carboxylase
		HMPREF1120_00351	21.31	4.77	8.00	Pyruvate carboxylase
	*GPM3*	HMPREF1120_06520	3.97	7.93	11.87	Phosphoglycerate mutase
	*TDH1-3*	HMPREF1120_04315			5.89	Glyceraldehyde-3-phosphate dehydrogenase
	*FBA1*	HMPREF1120_07847			5.39	Fructose-biphosphate aldolase class I
	*FBA2*	HMPREF1120_08620	4.28	4.32	14.22	Fructose-biphosphate aldolase class II
	*FBP1*	HMPREF1120_04809			5.54	Fructose-1,6-bisphosphatase
	*PGI1*	HMPREF1120_08503			2.91	Glucose-6-phosphate isomerase
Chitin synthase	*Chs 2*	HMPREF1120_06816			0.45	CHS2 Class I chitin synthase
	*Chs 1*	HMPREF1120_07981	9.51	6.13	7.01	CHS1 Class II chitin synthase
Chitin degradation	*ChiA*	HMPREF1120_03399	14.02	12.55	6.36	GPI anchored class III chitinase
	*ChiB*	HMPREF1120_06669			0.29	Class V chitinase
		HMPREF1120_04557			0.11	Chitinase
	*NagA*	HMPREF1120_06035	0.27	0.23		Extracellular N-acetyl-beta-glucosaminidase with a predicted role in chitin hydrolysis
1,3-beta-glucan synthesis and processing	*Cmg1*	HMPREF1120_05230			0.03	Glucans Putative exo-1,3-β-glucanase family (*GH 55*); related to Coniothyrium minitans exo-1,3-glucanase (*Cmg1*)
	*EglC*	HMPREF1120_00547			3.61	*Bgl2*-family of putative 1,3-β-transglucosylases (*GH 17*) proposed to be involved in connecting the emerging 1,3-β-glucan chains to the existing b-glucan network through 1,6-β-linkages; related to A. fumigatus *Bgt1*-family
	*Crf1*	HMPREF1120_00627	7.01	22.78	25.81	*Dfg5Crh1*-family of putative transglycosidases (*GH 16*); involved in crosslinking b-glucan and chitin; related to *ScCrh*
		HMPREF1120_02703			6.59	*Dfg5Crh*1-family of putative transglycosidases (*GH 16*); involved in crosslinking b-glucan and chitin; related to *ScCrh*
	*SunB*	HMPREF1120_06902			0.23	*Sun* family, involved in septation, possibly -glucosidase activity; related to *ScSun*-family Similarity
	*Kre6*	HMPREF1120_01614			0.44	Putative transglycosidase required for 1,6-β-glucan biosynthesis
	*CelA*	HMPREF1120_05299			0.21	Family similarity with cellulose synthases of the *GT 2* family. Putatively involved in 1,3-β-/1,4-β-glucan synthesis
	*Mlg1*	HMPREF1120_09051	8.63	60.97	20.25	Glucanases in *C. carbonum*, hydrolyze 1,3-β-/1,4-β-glucans
Other cell wall biosynthesis proteins		HMPREF1120_03513			0.31	Endo-mannanase family (*GH 76*) with a putative role in GPI-CWP incorporation; related to S. cerevisiae *Dfg5Crh1*
Ergosterol cell membrane	*ERG1*	HMPREF112_04761	10.40	5.79	6.18	Squalene monooxygenase
	*ERG24*	HMPREF1120_00726	11.96	8.91	4.52	Delta(14)-sterol reductase
	*ERG3*	HMPREF1120_04839	8.02	4.011		Delta(7)-sterol 5(6)-desaturase
	*ERG5*	HMPREF1120_06081	3.07	2.81	2.40	Delta(7)-sterol 5(6)-desaturase
	*ERG6*	HMPREF1120_06358	37.87	18.77	5.53	Sterol 24-C-methyltransferase *erg6*
	*MVD1*	HMPREF1120_03660	2.81	7.07	6.72	Diphosphomevalonate decarboxylase

We further conducted an inhibition test of DHN melanin production with tricyclazole on the wild-type and mutant strain. It induced an increase in melanin production but no depigmentation. Further melanin was excreted by the fungus into the medium. Interestingly, the mutant was able to take up melanin from the medium (**Supplementary Figure S2**). The mutant did not exhibit any changes in presence of tricyclazole. L-Dopa melanin production was successfully induced in the mutant by using liquid minimal medium containing L-Dopa. Finally the use of L-tyrosine in liquid minimal media did not induce melanin production in the mutant (**Supplementary Figure S2**).

#### Carbon Uptake

During growth on skin, WT *E. dermatitidis* activates the gluconeogenesis, glyoxylate and beta-oxidation pathways (Poyntner et al., 2016). In this work, WT2 and MT showed a similar trend. In the beta-oxidation pathway *POT1* (3-oxoacyl CoA thiolase), which cleaves 3-ketoacyl-CoA into acyl-CoA and acetyl-CoA, is enriched in the WT1U, WT2U and MTU. Acetyl-CoA is converted to oxaloacetate in the glyoxylate synthase pathway, where the key enzymes *Icl1* (isocitrate lyase) and *Cit2* (citrate synthase) are enriched in WT1U, WT2U and MTU, while *Aco1* (aconitase) is exclusively enriched in WT1U and WT2U. Oxaloacetate is then processed by the gluconeogenesis pathway to yield glucose. In *E. dermatitidis*, this happens with the help of *Mae1* and *Pyc1* which are upregulated in the wild-type and mutants during growth on skin. The next step involves *PCK* (phosphoenolpyruvate carboxykinase) which is upregulated in WT1 and MT. Further genes regulated along the gluconeogenesis pathway in WT1, WT2 and MT are phosphoglycerate mutase (*GPM3*) and fructose-biphosphate aldolase class II (*FBA2*), while glyceraldehyde-3-phosphate dehydrogenase (*TDH*) and fructose-biphosphate are upregulated only in the MT (**Table 1**).

#### Metal Acquisition

Iron uptake in *E. dermatitidis* happens over the siderophore pathway and through reduction of Fe^2+^ to Fe^3+^ (Poyntner et al., 2016). The enzymes responsible for the synthesis of siderophores *SidD*, *SidF*, *SidA* and *SidC* (Chen et al., 2014) and their transporter *Sit1*, are upregulated in all three skin experiments. Similarly, two genes related to the reductive uptake pathway, *Ftr1* and *Fet3*, are upregulated in WT1, WT2 and MT (**Table 2**).

**Table 2:** Significantly regulated genes involved in metal transport either in wild-type 1 (WT1), (Poyntner et al., 2016), wild-type 2 (WT2) and mutant (MT) or in all three experiments.

Category	Gene	Homolog	WT1	WT2	MT	Description	Reference
Iron transport	*Ftr1*	HMPREF1120_04509	6.76	64.20	53.35	High-affinity iron transporter	Stearman et al., 1996
	*Fet3*	HMPREF1120_04510		42.88	23.29	Ferrooxidoreductase Fet3	
	*SidF*	HMPREF1120_01438	4.55	3.58	5.95	Acetyl CoA:N6-hydroxylysine acetyl transferase	Chen et al., 2014
	*SidD*	HMPREF1120_01440	8.74	4.41	6.92	Nonribosomal peptide synthase Pes1	
	*SidA*	HMPREF1120_07635	7.34	4.25	8.73	L-ornithine N5-oxygenase	
	*SidC*	HMPREF1120_07636	2.46			Nonribosomal siderophore peptide synthase SidC	
	*Sit1*	HMPREF1120_01434	4.47	7.10		MFS transporter, SIT family, siderophore-iron: H+ symporter	Heymann et al., 2000; Petris, 2004
		HMPREF1120_02555	7.07	5.76	7.22	MFS transporter, SIT family, siderophore-iron: H+ symporter	
		HMPREF1120_07838	14.26	16.02	10.33	MFS transporter, SIT family, siderophore-iron: H+ symporter	
Copper pransport	*Crt*	HMPREF1120_00028			4.74	Copper transporter family	Petris, 2004
		HMPREF1120_05417			7.31	Copper transporter family	
		HMPREF1120_00028			4.72	Copper transporter	
	*Atx1*	HMPREF1120_03801			4.78	Heavy metal associated domain, Cu receptor	Lin et al., 1997

In contrast, genes related to copper transport from the environment into the cell, like *Crt* (Puig et al., 2002; Ding et al., 2014) and *Atx1*, are specifically upregulated in the mutant (**Table 2**). In *Cryptococcus neoformans Atx1*, a copper chaperone, is not only associated with copper transport but also with melaniniron uptake (Walton et al., 2005).

#### Environment Sensing

Among the genes responsible for the photoreception, only carotenoid oxygenase was downregulated in all three experiments, while the other genes were not consistently regulated (**Supplementary Table S1**). Among the genes involved in the MAPK pathway, *Ypld1*, which mediates the multistep phosphotransfer reaction is upregulated in the MT leading to the downregulation of *Ssk1* (Suppressor of Sensor Kinase HMPREF1120_04973). A homolog to *Tco2* (Sensory transduction histidine kinase, HMPREF1120_5233) is solely upregulated in the MT.

#### Cell Wall Regulation

Based on the annotation of the cell wall genes in *E. dermatitidis* (Chen et al., 2014) our results show that five out of nine chitin synthase genes and four out of eight chitin degradation genes are significantly regulated in the MT (**Table 1**), indicating that this strain is reorganizing more thoroughly its cell wall upon growth on skin than the WT. Among those genes one chitin synthase, *Chs1*, and one chitin degradation gene, *ChiA*, are also regulated in WT1 and WT2. The chitin degradation gene *NagA* is significantly downregulated in WT experiments but not in the MT (**Table 1**).

In the set of beta-glucan synthesis and processing genes, two genes are upregulated in all three skin experiments (**Table 1**), while four genes are downregulated only in the MT (*SunB*, *Kre6*, *CelA*, HMPREF1120_05230) and 2 genes are upregulated in the MT.

Finally, HMPREF1120_03513, a gene belonging to the family of endo-mannanase, with a putative role in glycosylphosphatidylinositol-bound cell-wall protein incorporation (Chen et al., 2014), is downregulated specifically in the MT (**Table 1**).

The production of ergosterol, the main component of fungal cell walls (Weete et al., 2010), increases in all three skin experiments. *ERG1*, *ERG24*, *ERG5*, *ERG6* and *MVD1* are all significantly upregulated in the WT and MT, while *ERG3* is upregulated only in the WT strains (**Table 1**).

#### Virulence Related Genes

Homologs of genes related to virulence mechanisms including adherence, signaling pathways, invasion and dimorphism reported in other pathogenic fungi were examined. Among the eight homologous genes related to dimorphism reported in Mayer et al. (Mayer et al., 2013) (Supplementary Material), only one member of the protease *Sap1-10* genes set is significantly downregulated in the MT (HMPREF1120_05067).

*Ssa1* is a member of the HSP70 family proteins reported to be expressed on the cell surface. It functions as a receptors for antimicrobial peptides in *Candida albicans* (Sun et al., 2010) and has an important role in endocytosis in epithelial cells. *E. dermatitidis* has seven known homologs of *Ssa1*. Four are significantly upregulated in the three experiments and one gene is upregulated only in WT1 and WT2 (Supplementary Material).

We further looked at the expression patterns of extracellular proteases. HMPREF1120_01991, a *S41* peptidase, is upregulated in both WT skin experiments but not in MT. The *S41* protease family is hypothesized to be involved in the basal fungal metabolism and to have occasionally developed specific characteristics connected to virulence (Muszewska et al., 2017). MT downregulated specifically a *S08* (HMPREF1120_08439, Cerevisin), *S09* (HMPREF1120_01940) and *S10* (HMPREF1120_5855) peptidases when growing skin. A DNase, (HMPREF1120_05403) is regulated both in the MT and the WT during skin infection. DNase were previously linked to virulence in *E. dermatitidis*, *Cryptococcus neoformans* and *gattii* (Sánchez and Colom, 2010; Sav et al., 2016).

#### Secondary Metabolites

Among the genes involved in secondary metabolites synthesis (Poyntner et al., 2016), a homolog of dimethylallyl tryptophan synthase (HMPREF1120_01968, **Table 1**), a gene involved in the synthesis of ergot alkaloid, was upregulated in the MT experiment. Linear gramicidin synthetase subunit C (HMPREF1120_00598), a non-ribosomal peptide synthetase involved (NRPS), was upregulated in all skin experiments.

## Discussion

In this work the CRISPR/Cas9 gene knockout method has been successfully used in *E. dermatitidis*. The targeted gene, *PKS1*, which is required for the melanin production of the DHN melanin pathway (Feng et al., 2001), was disrupted successfully. The MT and WT strains were inoculated and cultured on vital human skin using identical *ex vivo* culture conditions and were monitored microscopically and transcriptomically. While both strains grew at a similar rate on the nylon membrane control, the WT proliferated significantly faster than the MT on the *ex vivo* skin models. Macroscopic findings were confirmed by HE microscopy and in the HE-stained sections it was clearly visible that the WT was able to infect and disintegrate the skin and invade into deeper parts of the dermis, while the MT was only colonizing the surface of the epidermis. The skin model inoculated with the MT maintained its vital structure compared to the WT, where separation of epidermis and dermis as well as pyknotic keratinocytes were visible. This indicates that the absence of melanin reduced the ability of the MT to colonize the skin. The changes in transcriptional landscapes between WT and MT in the control and in the skin experiments were minimal. We looked therefore at differences in the enriched categories of the differentially expressed genes between the skin experiment and control of the WT or MT for each strain separately (WT1, WT2, and MT).

From the transcriptome point of view, the main differences between the mutant and wild-type were seen in the regulation of copper transporters. It is known that melanin increases copper biosorption (Gadd and de Rome, 1988; Glass et al., 2014), probably increasing the copper concentration in vicinity of the WT membrane. Therefore the MT might upregulate the copper transporters to compensate for the reduced copper concentration in the proximity of the fungal cell. Additionally copper is a crucial component of melanin synthesizing enzymes like laccase and tyrosinase (Li et al., 2016). The nitrogen acquisition pathway was also impacted by the disruption of *PKS1*. In MT, four peptidases are downregulated in the *ex vivo* skin model experiment, among which two are metallopeptidases (*M28*) that can be activated by divalent cations including copper. Further the lack of melanin did impact the regulation of cell-wall components like chitin and b-glucan as well as the ergosterol biosynthesis indicating a reorganization of the cell wall in the MT. The enrichment in MCM complex is only found in the WT strains, supporting the reduced growth seen macroscopically and microscopically in the MT experiment compared to the WT experiments.

In all three experiments we find negative regulation of the genes involved in the synthesis of DHN and L-Dopa melanin. The DHN pathway seems to be crucial for the invasion of the skin model as the mutation of the *PKS1* gene of the DHN pathway leads to completely different microscopic and macroscopic pictures. L-Dopa added to minimal liquid media induced the production of melanin in MT, in line with similar experiments (Paolo et al., 2006).

The L-Tyrosine pathway was upregulated even in the melanin deficient mutant but did not lead to a blackening of the mutant on skin. The addition of L-tyrosine in liquid media did not lead to pyomelanin production in the mutant either. The reason might be that the phenolic homogentisate acid, which is a precursor of the pyomelanin in this pathway, is also degraded to fumarate and acetoacetate (Keller et al., 2011). One of the genes responsible for the degradation (*fahA*) is upregulated 45 times in the mutant (**Table 1**). This indicates that the degradation products of L-tyrosine are primarily used in the Krebs cycle and not for pyomelanin production. The neurotropism of *E. dermatitidis* may be associated with its ability to use L-tyrosine as an energy source. *E. dermatitidis* and other black yeast species were isolated from mono- and poly-aromatic compounds rich places such as creosote covered railway sleepers (Döğen et al., 2013a) or toxic hydrocarbons (Prenafeta-Boldú et al., 2006; Zhao et al., 2010; Blasi et al., 2016) and seem to be able to grow on aromatic substances. A link between the capabilities of fungal strains to degrade contaminants and being neurotropic agents for warm-blooded vertebrates is hypothesized (Prenafeta-Boldú et al., 2006).

The difference in skin invasion between the WT and MT are probably due to the disruption of DHN melanin production. Lack of skin invasiveness of an albino fungal strain compared to the pigmented one, has previously been reported in an experimental rat model with sporotrichosis (Barros et al., 2011). Similarly, DHN melanin extracted from the black yeast *Aureobasidium pullulans*, was shown to significantly inhibit the proliferation of cultivated human keratinocytes (Blinova et al., 2003). Melanin may play various roles in host intrusion. It is involved in the formation of the appressorium, a dome-shaped cell that applies physical force to rupture and invade host plant cells (Howard and Ferrari, 1989). While appressoria are not reported to be formed by zoopathogenic fungi, it was shown that melanised *E. dermatitidis* hyphae grew faster through thick agar than their albino counterparts (Brush and Money, 1999). Recently, it was shown that DHN melanin synthesized by the banana pathogen Mycosphaerella fijiensis can photogenerate singlet molecular oxygen, which may be involved in the damaging of the plant (Beltrán-García et al., 2014).

In our study the category of Lon Protease S16, which among other contributes to pathogenicity of bacteria and fungi (Takaya et al., 2003; Breidenstein et al., 2012; Cui et al., 2015; Li et al., 2015), was exclusively enriched in the set of upregulated genes in the WT experiments. Further a putatively excreted serine protease (HMPREF1120_01901, *S41*) is upregulated only in the WT during growth on skin and three excreted serine proteases were exclusively downregulated in the mutant during skin growth (HMPREF1120_01940 (*S09*), HMPREF1120_05855 (*S10*), HMPREF1120_08349 (*S08*, Cerevisin)). In humans, physiological melanin uptake by endocytosis, a process to protect the nucleus and DNA from influences of UV irradiation (Ando et al., 2012), occurs after activation of the protease-activated receptor-2 (*PAR*-2) (Seiberg et al., 2000a). The *PAR-2* receptor is activated by a serine protease (Seiberg et al., 2000a), while serine protease inhibition is accompanied with reduced melanin transfer and skin lightning (Seiberg et al., 2000b). Proteases from the skin pathogens *Serratia marcescens* and *Propionibacterium acnes* were shown to activate *PAR-2* (Koziel and Potempa, 2013), as did various serine proteases in a murine model (Andoh et al., 2012). *PAR-2* activation by trypsin, *SLIGRL* or *SLIGKV* increases the ability of keratinocytes to ingest fluorescently labeled microspheres or *E. coli* K-12 bioparticles (Sharlow et al., 2000). Interestingly, two recent studies showed that melanin absorbed by keratinocytes is located in non-degradative compartments (Correia et al., 2018; Hurbain et al., 2018).

Given the facts that human keratinocytes absorb exogeneous melanin (Huang et al., 2017) and that lung epithelial cells uptake of *PKSP* mutant conidia from *Aspergillus fumigatus* was much lower than of its melanised counterpart (Amin et al., 2014), it can be hypothesized that the fungus uses the melanin uptake system to either invade the keratinocytes or channels virulence factors into it in a “trojan horse” manner. Hence, lack of melanin along with the serine protease crucial for endocytosis may have accounted for the lack of invasion and virulence seen with the MT fungus. The use of endocytosis to promote infection in a “trojan horse” manner has been proposed for fungi, bacteria, virus and even bacteriophage (Peluso et al., 1985; Nguyen et al., 2017; Santiago-Tirado and Doering, 2017). More specifically, the yeast *Candida albicans* secretes aspartic proteases that are internalized to endosomes and lysosomes in epithelial cells and subsequently trigger apoptosis (Wu et al., 2013).

Overall the albino and the wild-type strains exhibit similar transcriptional landscapes but show profound differences in cutaneous pathogenicity with regard to invasiveness and skin desintegration in their microscopic appearance. The lack of melanin impacts mainly the fungus’ cell wall structures, the copper homeostasis, protein degradation and ultimately the speed of growth and invasiveness of the mutant in the *ex vivo* skin culture model, indicating that melanin, while not being strictly necessary during the first phase of the skin colonization, is crucial for invading the skin. This finding is in line with experiments showing that albino strains of *E. dermatitidis* is associated with reduced case fatality rates in mice (Dixon et al., 1987; Feng et al., 2001). Further the changes in the copper homeostasis indicate that this metal plays an important role in the first phase of infection.

## Conclusion

In conclusion, the usage of CRISPR/Cas9 and RNA sequencing allowed us to knock-out the *PKS1* gene, disrupt melanin production and assess the impact on the virulence of the fungus. We could show that melanin plays a key role with regard to the fungus’ virulence by facilitating skin invasion and disintegration.

In contrast to our *ex vivo* skin model, fungi involved in human infections face host defense mechanisms, among others the phagocytosis by macrophages and oxidative burst (Lorenz et al., 2004). In order to better understand the relevance of melanin in oxidative stress resistance, we plan to grow the wild-type and the mutant under oxidative conditions and compare their transcriptional response and growth rate. To understand if human cells can distinguish between fungal and human melanin, we further plan to investigate the uptake mechanism of fungal melanin into the human skin cells and the impact of melanin on the first human immune response.

## Author Contributions

HT, CP, and KS designed the experiments. UM prepared the skin models. CP carried out the lab work and the microscopy. HT developed bioinformatic tools for data analysis. HT and CP analyzed the data. CP, HT, KS, and UM wrote the manuscript. All authors have read and approved the final manuscript.

## Conflict of Interest Statement

The authors declare that the research was conducted in the absence of any commercial or financial relationships that could be construed as a potential conflict of interest.

## Footnotes

1 www.vbcf.ac.at

## Supplementary Material

FIGURE S1 PCA based on the count vector obtained from feature Counts for the 15 RNA sequencing runs. MT stands for mutant, WT for wild-type, CT is the control experiment and Skin is the skin experiment. Skin dataset with the Old suffix are from the Poyntner et al., (2016) paper on skin infection.

FIGURE S2 Malt extract agar plates with either tricyclazole (50 mg/L, first row) inoculated for 10 days at 37°C with the mutant (left plate), wild-type (middle plate)or mutant and wild-type together. Liquid minimal medium with L-Dopa (1 mM, second row) or L-tyrosine (1 mM, third row) inoculated with the wild-type or mutant did show color change for the mutant with L-Dopa but not with L-tyrosine.

TABLE S1 Table containing supplementary data on cell wall related genes, sgRNAs, mapping, featureCount, functional annotation, regulated virulence factors, putative expressed serine proteases, up and down regulated genes skin vs. controls, upregulated genes in WT vs. MT and dataset of enriched GO terms.
