# Supplementary figures and images for: Transcriptome Study of an Exophiala dermatitidis PKS1 Mutant on an ex Vivo Skin Model: Is Melanin Important for Infection?
Source: Front Microbiol. 2018 Jul 3;9:1457. doi: 10.3389/fmicb.2018.01457 (PMC6037837; doi:10.3389/fmicb.2018.01457)

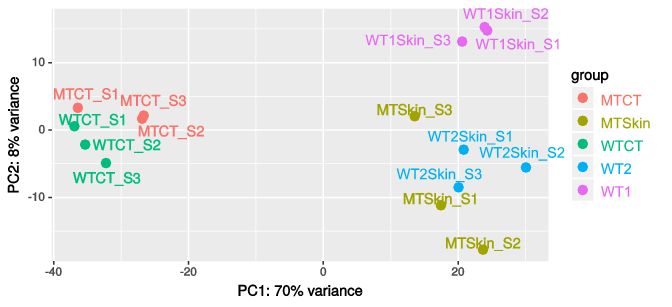

Supplement: FIGURE S1 — PCA based on the count vector obtained from feature Counts for the 15 RNA sequencing runs. MT stands for mutant, WT for wild-type, CT is the control experiment and Skin is the skin experiment. Skin dataset with the Old suffix are from the Poyntner et al., (2016) paper on skin infection. [file Image_1.JPEG]

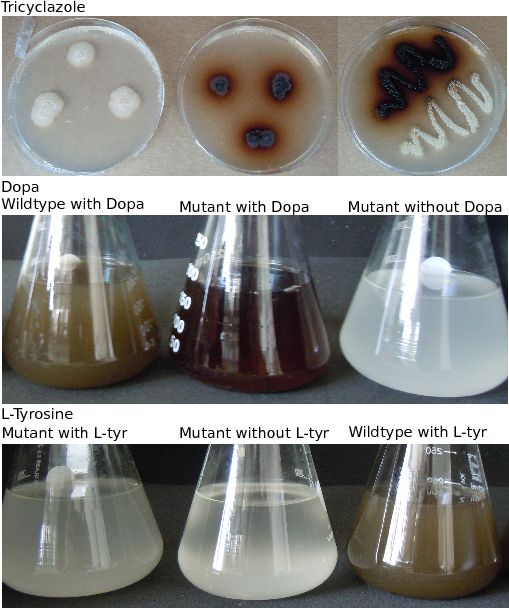

Supplement: FIGURE S2 — Malt extract agar plates with either tricyclazole (50 mg/L, first row) inoculated for 10 days at 37°C with the mutant (left plate), wild-type (middle plate)or mutant and wild-type together. Liquid minimal medium with L-Dopa (1 mM, second row) or L-tyrosine (1 mM, third row) inoculated with the wild-type or mutant did show color change for the mutant with L-Dopa but not with L-tyrosine. [file Image_2.JPEG]
